# Recent Trends in System-Scale Integrative Approaches for Discovering Protective Antigens Against Mycobacterial Pathogens

**DOI:** 10.3389/fgene.2018.00572

**Published:** 2018-11-27

**Authors:** Aarti Rana, Shweta Thakur, Girish Kumar, Yusuf Akhter

**Affiliations:** ^1^School of Life Sciences, Central University of Himachal Pradesh, Shahpur, India; ^2^Department of Biotechnology, Babasaheb Bhimrao Ambedkar University, Lucknow, India

**Keywords:** *Mycobacterium*, vaccine, diagnostic markers, reverse vaccinology, antigen discovery

## Abstract

Mycobacterial infections are one of the deadliest infectious diseases still posing a major health burden worldwide. The battle against these pathogens needs to focus on novel approaches and key interventions. In recent times, availability of genome scale data has revolutionized the fields of computational biology and immunoproteomics. Here, we summarize the cutting-edge ‘omics’ technologies and innovative system scale strategies exploited to mine the available data. These may be targeted using high-throughput technologies to expedite the identification of novel antigenic candidates for the rational next generation vaccines and serodiagnostic development against mycobacterial pathogens for which traditional methods have been failing.

## Introduction

Despite the massive advancements over the years in the field of effective clinical interventions, a big number of people in the developing countries still suffer from an enormous burden of contagious diseases. Various pathogens such as viruses, bacteria, parasites and fungi are responsible for these widespread infections (Janeway et al., 2001). Over the past decade, among them, mycobacteria are recognized as the most common cause of serious illness and deaths globally (WHO, 2016). The mycobacterial pathogens continually present us with ongoing threats to human and animal health and challenge our endeavors to obstruct and control infectious diseases. Among these, *Mycobacterium tuberculosis* (*Mtb*), *M. leprae, M. bovis* and *M. avium subsp. paratuberculosis* (MAP) are the four largely known and well established mycobacterial species that can cause a variety of dreadful infectious diseases, such as tuberculosis (TB), leprosy in humans and paratuberculosis in animals (Hoffmann et al., 2018). The overall disease burden posed by these microbes has been constantly on the rise and hence, it is crucial to stop their spread by developing sensitive diagnostic tools for their early detection and design effective vaccines to generate long-term immunoprotection against such infections.

## Commonly Available Prophylactic Health Interventions Against Mycobacterial Infections

Foundation of modern medicine has been laid down on valuable anti-infective drugs now in use. However, the rapid evolution of antibiotic resistance has now become a limitingcondition that may impose a considerable economic burden and endanger the efficacy of antibiotics for the control of many infectious diseases (Fair and Tor, 2014). Antibiotic resistance is a disaster which arises due to the excessive exploitation of medications, as well as a lack of new effective vaccines manufactured by the pharmaceutical industry (Ventola, 2015). Therefore, discovering new prophylactic treatments to remedy the infectious diseases has been a major focus of modern medicine. Below, in the next subsections, currently available vaccine candidates and their safety issues have been discussed.

### Vaccines

**FIGURE 1:**
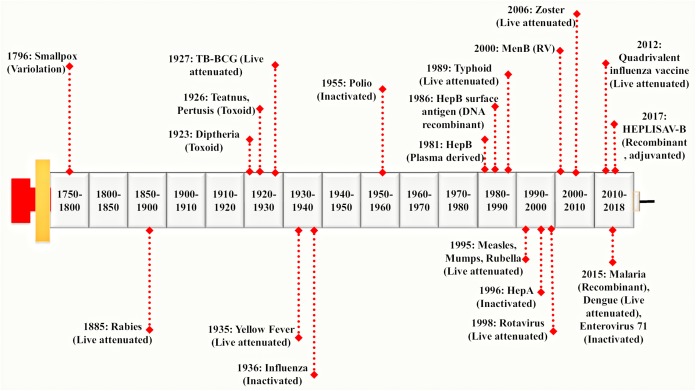
Evolution of vaccine development processes: Vaccine development was pioneered by Edward Jenner. He discovered a working vaccine against small pox in 1796 derived by variolation and further work was continued by Louis Pasteur. He has discovered a live attenuated vaccine against Rabies virus in 1885 considered to be one of the 1st generation vaccines. It was followed by a genomic revolution and in the post-genomic era, mankind witnessed the modern sequencing techniques. In early 21st century, Rappuoli introduced Reverse Vaccinology (RV) approach which provided a foundation to the development of 2nd generation vaccines (Rappuoli et al., 2016). Since then, advances in various ‘omics-based’ approaches together with RV led to the development of a much more advanced 3rd generation of vaccines in the present times. Different vaccines derived from variolation, live attenuated, inactivated, toxoid, DNA recombinant have been shown in the timeline.

Vaccines were used extensively before the antibiotics became accessible. Vaccination proves to be the most successful available strategy of an integrated prevention/therapeutic toolkit. It has significantly reduced the prevalence of a variety of infectious diseases such as bacterial and viral infections. It has slowed down the rate of development of resistant strains thereby preventing the further spread of several devastating infections globally (Andre et al., 2008; Rana and Akhter, 2016). A vaccine represents a biological formulation which upon administration to a given population can generate life time’s immunity against a particular disease (Mohan et al., 2013). First generation vaccines were developed using attenuated or inactivated strains of microbial pathogens. These have been reported as efficient for inciting both humoral and cellular immune responses (Seder and Hill, 2000). The second generation vaccine is composed of pathogen-derived purified components (devoid of the factors responsible for infection) instead of the whole microbial cells. These have been developed using novel recombinant proteins and DNA molecules (rDNA technology) as well as non-virulent but immunoprotective forms of microbial pathogens. The high-throughput sequencing and availability of complete genomic information have paved the way to a new ‘third generation’ of the vaccines (Seib et al., 2009). On vaccine administration, the vaccinated individual’s immune system encounters antigens expressed by disease-causing foreign pathogen and remembers it in form of immunological memory. This immunological memory, when encounters the real microbe expressing those antigens, there is production and activation of highly specific memory T lymphocytes, B lymphocytes and natural killer cells (Ratajczak et al., 2018). This rapidly generates an effective immune response against the microbial pathogen (Ottenhoff and Kaufmann, 2012). Hence, the most important job of vaccines is to expose the vaccinated individuals with much milder and non-virulent pathogenic antigens to generate immunological memory without actually causing the disease. A brief history of major breakthroughs in vaccine development has been illustrated in Figure 1.

The most commonly used first generation vaccine against the mycobacterial pathogens is Bacillus Calmette-Guerin (BCG). It is composed of attenuated (non-virulent) strains of *M. bovis.* In the following subsections, we are summarizing the current use and protection status provided by the BCG vaccine.

#### The BCG Vaccine

Currently, BCG is the only TB vaccine which is inexpensive, safe and readily available. It is composed of live attenuated strains of *M. bovis* (Lahey and Von Reyn, 2016). It induces an immune response against the *Mycobacterium* without actually causing the disease (Trunz et al., 2006). Since it is cheap, it is considered as the most economical way to provide protection to millions of children against TB and leprosy globally (Zwerling et al., 2011). Although the BCG vaccine is one of the oldest extensively used vaccine, it may not be presented as the most successful available strategy. BCG has been reported with incomplete protection against *Mtb* and *M. leprae* infection. Over previous decades, different clinical trials and epidemiological studies have been conducted to evaluate the efficacy of BCG in many countries (Trunz et al., 2006). Studies showed that BCG vaccination provides 60–80% protective efficacy to prevent dissemination in children who were otherwise suffering from TB, meningitis, miliary disease and pulmonary TB (Roy et al., 2014). Despite its widespread use, BCG vaccine has been reported to be less effective in TB endemic zones (Brandt et al., 2002).

In the case of leprosy, numerous attempts have been made for the development of a highly specific vaccine against leprosy but still, the efforts have not met with complete success. Currently, the only licensed vaccine administered for protection against *M. leprae* is the BCG vaccine. This protection has been reported to wane over time as is the case with BCG generated protection against TB infection (Duthie et al., 2011, 2012). Therefore, there is a great need for the discovery of ideal vaccines that may provide better protective efficacy against TB and leprosy. To better understand the mechanistic details about the failures of the BCG vaccine, in the following subsections safety issues, diversity among various BCG strains and their molecular evolution have been discussed.

#### Safety Issues and Variability in the Efficacy of BCG Vaccine

Bacillus Calmette-Guerin vaccine has been used as a “gold standard” because of its cosmopolitan availability and cost-effectiveness. BCG side-effects are usually very rare and include inflammation at the site of injection among vaccinated individuals (Rowland and McShane, 2011). Another important BCG vaccine safety issue for consideration is its efficacy among the immune-compromised individuals. In the HIV-positive children, an increased risk of diseases was reported which ultimately forced the WHO to put forward the restriction on BCG vaccine administration among HIV-positive children (Brennan and Thole, 2012). The HIV infected immune-compromised individuals administered with BCG vaccine have been observed with an onset of BCG disease because of the primary immunodeficiency. As BCG activates the CD4+ T cells (HIV targeted cells), it may increase the susceptibility of children to HIV infection and accelerate HIV disease progression (Santema et al., 2013). A number of reports have been cited in the literature demonstrating the wide-ranging variability observed in the BCG efficacy. A majority of the reports suggest a nearly 80% BCG efficacy while some of the reports conclude that BCG is completely ineffective (Mangtani et al., 2013). Some studies have reported that BCG administration to children may result in mycobacterial dissemination to various other organs also, which may prove lethal. Moreover, BCG fails to generate complete protection in a patient suffering from adult pulmonary TB (Kernodle, 2010). Some of the major potential reasons responsible for the observed changes in the efficiency of BCG are covered in the following subsections. These include the genetic variability within available versions of attenuated BCG strains and the genetic immuno-polymorphism among the human populations on which the vaccine has been administered. Prior exposure to mycobacterial strains (including environmental mycobacteria) affecting the outcomes of vaccine trials has also been discussed briefly.

##### BCG strain variation

The *M. bovis* BCG parent strain was originally developed in 1921 at the Pasteur Institute. The attenuated form of *M. bovis* was derived through the serial passage of virulent *Mycobacterium* isolated from a cow suffering from tuberculous mastitis. This attenuated strain was disseminated to several laboratories and developed into different sub-strains possessing different characteristics worldwide (Oettinger et al., 1999). These include evolutionarily early BCG strain: Japan and the evolutionarily late BCG strains: Connaught, Glaxo, Pasteur, Danish and Tice. Some of the commonly used BCG strains for the development of BCG vaccine have been mentioned in Table 1 (Ritz and Curtis, 2009; World Health Organization, Immunization, Vaccines and Biologicals Department, 2012; da Costa et al., 2014).

**Table 1:** Different BCG vaccine strains available worldwide.

Sr. No.	BCG vaccine strains	Lost RD regions	Reference
[1]	BCG Tokyo/Japan	RD1	Tang et al., 2008; Lin et al., 2011
[2]	BCG Danish/Denmark	RD1, RD2	Shi et al., 2010; Rahman et al., 2012
[3]	BCG China/Shanghai	RD1, RD2	Deng et al., 2012
[4]	BCG Prague	RD1, RD2	Reece et al., 2011; Farinacci et al., 2012
[5]	BCG Pasteur	RD1, RD2, RD14, nRD18	Tang et al., 2008; Sali et al., 2010
[6]	BCG Tice	RD1, RD2, nRD18	Hoft et al., 2008; Tullius et al., 2008
[7]	BCG Glaxo	RD1, RD2	Zhang et al., 2013
[8]	BCG Connaught	RD15, RD18	Witjes et al., 2016


*^∗^RD represents a region of difference.*

##### Diversified genetic make-up of the test individuals

A vaccine’s efficacy is more or less dependent upon the genetic make-up of the test population. The variation in the form of single nucleotide polymorphisms (SNPs) in the test population genomes can affect their susceptibility to disease and its outcome. It also governs the protective immune response generated by a particular vaccine (MacDonald and Izzo, 2015). The immune response may vary from complete protection to no protection at all. A study demonstrating the genetic variation effect on BCG vaccine efficacy has reported dissemination of disease after BCG administration in patients with mutated IFN-γ receptors (Döffinger et al., 2002). The earlier conducted BCG vaccination clinical trials have shown consistently a suitable immune response against *Mtb* in UK infants, but on the other hand offered a very little to nil protection among infants of Malawi. This noticeable population variance in the generated immune response against BCG vaccine indicates that it might not be possible to offer equal immunity to the infants from different countries (Lalor et al., 2011). The BCG administered Malawian infants were found to develop T cell immune response with an early cytokine profile that was found to be completely different from that generated among the BCG vaccinated UK infants. This was characterized by the presence of a large population of antigen-specific IFNγ dominated Th1 cells (Lalor et al., 2011). While another study conducted on BCG-vaccinated infants from Indonesia recognized marked induction of not only IFNγ but also of IL-5 and IL-13 in contrast with the findings from the Malawian and UK infants (Djuardi et al., 2010). Hence, the different cytokine bio-signatures generated in the form of immune responses following BCG vaccination in population with differences in their genetic makeup could be attributed as one of the important reasons for observed variability in efficacy of BCG vaccine (Lalor et al., 2009; Dockrell et al., 2012; Kollmann, 2013). Moreover, individuals with observed mutational changes in genes susceptible to a particular disease become highly vulnerable to various other commonly found mycobacterial infections from the environment (Döffinger et al., 2002). Therefore, monitoring of vaccine trials, with appropriate biomarker measurements and genomic diversity of the test individuals must be considered as there is no homogenous population distribution in the world. Therefore, the criteria to carry out a clinical trial for any antimycobacterial vaccine candidate should be laid down carefully.

##### Pre-exposure to the pathogens and related environmental mycobacteria

Another significant issue of huge importance to be considered while conducting BCG efficacy tests is an individual’s pre-exposure to the pathogen. An individual with a pre-exposure to a particular antigen has a different immune response as compared to someone with no earlier exposure to the antigen. For instance, the children in countries with TB and leprosy-endemic zones have a pre-exposure to *Mtb* and *M. leprae.* During various TB and leprosy eradication programs, a huge variability has been observed in the generated immune response on BCG administration among children (Andersen and Woodworth, 2014). Additionally, exposure to the environmental mycobacteria including the non-tuberculous mycobacteria (NTM) found in water and soil generates cross-reactive immune responses which further blocks the BCG activity (Demangel et al., 2005; Halstrom et al., 2015). Hence, a highly efficient and effective vaccine should thus be passed through extremely stringent clinical testing which should consider only those individuals with no pathogen pre-exposure (Mangtani et al., 2013).

## Conventional Approaches to Vaccine Development

In 1880, Louis Pasteur when administered *Pasteurella septica* in chickens, it generated protection against fresh virulent bacterium in the chickens. This demonstrated that the pathogenic bacteria lost disease-causing properties and got completely attenuated (changed into the non-virulent but immunoprotective forms) (Movahedi and Hampson, 2008). Subsequently, a year later, he prepared a vaccine against anthrax using attenuated forms of *Bacillus anthracis*. His novel approach was further utilized by the scientific community to form the foundation of vaccine discovery. It consists of isolating the pathogen, its attenuation followed by administration of the antigenic pathogen. This approach has allowed the development of vaccines against prevalent diseases in the twentieth century (Serruto and Rappuoli, 2006; Meeusen et al., 2007; Movahedi and Hampson, 2008). The conventionally developed vaccine is based on 2 approaches: attenuating the targeted microbial pathogens *in vitro* by growing it in growth media several times to obtain a viable non-virulent strain and identifying highly specific potential antigenic components from microbial pathogens (Sette and Rappuoli, 2010). The immunodominant antigenic components of the targeted pathogens are identified by various sera-based methods and molecular genetics based methods. These conventionally available methods are very cumbersome, extremely slow and costly. Moreover, these methods can only be used to identify the highly abundant antigenic components which can be extracted in enough quantities appropriate for vaccine development (Bagnoli et al., 2011). Since the biological methods needed to isolate such components are poor in number, it generally takes decades to identify suitable antigenic molecules for vaccine development. The total number of identified potential immunogens to be used in vaccine development is extremely poor. It is documented that only 25 infections have licensed vaccines (WHO, 2012). These conventional approaches also fail when the microbial pathogens fail to grow in laboratory conditions on available supplemented/not supplemented artificial media (Donati and Rappuoli, 2013).

## Current Status of Known Biomarkers for Diagnostic Assays

In order to completely control and eradicate mycobacterial infections globally, accurate diagnosis followed by effective treatment is required. However, there are no gold standard diagnostic tests available against these mycobacterial infections. The available detection tools lack specificity and accuracy. Among the available diagnostic tools for *Mtb* detection, the tuberculin skin test (TST; standard is the Mantoux test) and interferon (IFN-γ) release assay (IGRA) are widely used. These both are indirect markers for the detection of *Mtb* infection and measure a cellular immune response to *Mtb*. Some of the challenges faced by these tools include incompetency to distinguish between active and latent TB, failure to differentiate reinfection from reactivation and poor sensitivity among immunocompromised patients (Pai et al., 2014). In TST, a delayed type 4 hypersensitivity reaction is generated when the purified protein derivative (PPD) obtained from *Mtb* is injected into the patient. It generally takes 48–72 h for obtaining the final results. This delay may mislay the patient’s compliance and exposure. In addition, the TST as well as some other newly developed serological tests, fail to distinguish between exposure to infectious *Mtb* and other environmental NTM. Hence, the performance of these diagnostic tools is continuously deteriorating and cannot be relied upon (Doan et al., 2017).

Currently, better serodiagnostic assays with high specificity for pathogenic mycobacterial infections and more sensitive than the available diagnostic tools are needed. One of the newly developed methods for the rapid detection of *Mtb* includes a nucleic acid amplification assay (NAAA) which targets the insertion sequence (IS) 6110 sequence from *Mtb*. It combines two PCR techniques: nested polymerase chain reaction (Nested PCR) and real-time polymerase chain reaction (Real-time PCR) in a single tube. The nucleic acid amplification test IS6110 has shown high levels of sensitivity to detect the presence of *Mtb*. One-tube nested RT-PCR is 100 times more sensitive in comparison to conventional RT-PCR (Choi et al., 2014). In another study, the culture and mpt64RT-PCR demonstrated the same sensitivity (90.3%) in sputum samples. While, mpt64RT-PCR recorded 98.6% specificity in comparison to culture (99.4%) and smear microscopy (99.7%). Hence, this modern day molecular technique NAAA can be utilized in routine laboratories enabling quick and specific TB detection within 5 h (Laux da Costa et al., 2015; Watanabe Pinhata et al., 2015).

In leprosy, the conventional diagnostic tools are usually dependent upon histopathology and bacillary counts of skin smears. Since *M. leprae* presents tropism for the skin (macrophages) and peripheral nerves (Schwann cells), the slit-skin smear (SSS) still remains the gold standard technique of choice for leprosy diagnosis. Serological tests detecting IgM antibodies against phenolic glycolipid-I (PGL-I; *M. leprae* cell surface antigen) and IFN-gamma releasing assays (IGRA) detecting IFN-gamma production are also being widely used for diagnosis of *M. leprae*. These classical methods have been found incompetent to distinguish the active disease from a latent form of *M. leprae* infection and are inefficient to diagnose the paucibacillary clinical forms of Hansen’s disease. Among the modern-day molecular techniques, especially PCR has emerged as an alternative tool for molecular diagnosis among the hard to diagnose cases of leprosy (neural, paucibacillary and indeterminate leprosy). In fact, the advances in *M. leprae* structural and functional genomics has allowed the development of highly specific PCR-based gene amplification assays for early rapid *M. leprae* DNA detection with high sensitivity. PCR has also proved useful in the *M. leprae* viability determination, identification of routes of transmission and leprosy drug resistance (Geluk et al., 2012; Martinez et al., 2014; Soto and Muñoz, 2015; Maltempe et al., 2016).

In case of Crohn’s disease, the MAP can be detected in infected animal’s milk samples *via* culture, enzyme-linked immunosorbent assay (ELISA) (Sorge et al., 2011), immunomagnetic separation (IMS) and PCR. For the detection of subclinical MAP infections, various serological methods like agar gel immunodiffusion, complement fixation and ELISA methods have been widely used.

Numerous epidemiological studies are still being carried out to find a reliable molecular method for the rapid and accurate detection of paratuberculosis from clinical samples. These include: real-time PCR, mycobacteria interspersed repetitive units (MIRU) typing, variable number tandem repeat (VNTR) typing, immunomagnetic separation-PCR (IMS-PCR), nested PCR, pulsed-field gel electrophoresis (PFGE), multiplex PCR and IS900 restriction fragment length polymorphism (RFLP) (McKenna et al., 2005; Romano et al., 2005; GÜMÜŞSOY et al., 2015).

In recent times, a number of potential diagnostic biomarkers have also been identified and are under study against mycobacterial infections. The recombinant proteins generated through a combination of secretory proteins from *Mtb*, Hsp16.3/ESAT6 and Ag85B-Hsp16.3/ESAT6 has been identified as highly potentially antigenic which may be targeted as serodiagnostic biomarkers (Zhang et al., 2015). These may represent the preliminary screening antigens against active TB. *Mtb* antigens, Rv1681 (Pollock et al., 2013), Rv0444c, Rv3692, and Rv2031c proteins (Zhang et al., 2012) have also been reported with potentials of diagnostic utility and hence these may be exploited as anti-TB biomarkers. The host or pathogen-specific biomarkers in recent times, which remained under investigation for the detection of mycobacterial pathogens, are listed in Table 2.

**Table 2:** Diagnostic biomarkers against mycobacterial pathogens.

Sr. No.	Biomarker	Reference
[1]	**Chemokines and Cytokines**	Wassie et al., 2008; Ruhwald et al., 2011; Mihret and Abebe, 2013
	IP-10 IL-4/IL-4δ2, IL-4/IFN-γ	
[2]	**Antigens for immunological assays**	
	HBHA protein	Hougardy et al., 2007
	DosR proteins	Riaño et al., 2012
	LAM	Lawn et al., 2013
	Antigen 85	Kashyap et al., 2007
	Rv1681	Pollock et al., 2013
	Hsp16.3, ESAT6	Zhang et al., 2015
	Rv2031c, Rv3692, Rv0444c	Zhang et al., 2012
	Rv0256c	Parveen et al., 2003
	PPE18	Bhat et al., 2012
	PPE25/PPE41	Choudhary et al., 2003; Tundup et al., 2008
	Rv1168c	Khan et al., 2008
	Isocitrate dehydrogenases	Banerjee et al., 2004
	Hsp60/65	Bodzek et al., 2014
	*Mycobacterium bovis* BCG r32-kDa	Priya et al., 2010
	Rv1513, Rv1973, Rv3738c	Yuan and Xu, 2017
	ESAT-6, CFP-10	Goletti et al., 2016


## An Analytical View of Modern Methodologies That Can Be Used for Efficient Antigen Discovery Against Mycobacterial Pathogens

With the complete sequencing of the human genome, a new era of systems biology known as ‘omics’ technology has emerged. The ‘omics’ technologies represent a holistic view of different molecules that constitute a cell of an organism. They are primarily aimed to explore genes under genomics, protein coding mRNA and non-protein coding RNA under transcriptomics, proteins under proteomics and metabolites under metabolomics in a specific biological sample (Horgan and Kenny, 2011; Tripathi et al., 2017). Currently, prevalent ‘omics’ technologies combined with advanced bioinformatics are constantly putting their efforts to unveil the mechanisms behind molecular pathogenesis of infecting microbes, which may further help us to devise treatment strategies against them. Employing these approaches to vaccine development could actually transform the very expensive purely experimental study of antigen discovery into a cost-effective theoretical and computational one. This scenario will definitely help in enhancing the prospects for novel antigen discovery by selecting the immunodominant epitopes for their use as prime vaccine candidates. Contributions made by various high-throughput technologies are discussed in further subsections.

### Genomics

Genomics may be described as a comprehensive analysis of an organism’s complete genome. The genome represents the complete set of DNA/genes (coding and non-coding) present in a cell or organism. There are approximately 3.2 billion bases and an estimated 20000 protein coding genes in humans. Traditionally, genes were analyzed individually but with the advent of microarray technology, genome-wide differential expression studies are made possible in recent years. DNA microarrays measure the subtle differences among DNA sequences (genetic variations) like small-scale insertion/deletions, polymorphic repetitive elements, SNPs and microsatellite variation among different individuals. The most common type of genetic variation is single nucleotide polymorphisms (SNPs). SNP occurs when one nucleotide in the genome is substituted for another and differs between members of the same species (Horgan and Kenny, 2011). This change results in an alternative codon and hence different amino acid which may be of particular interest when associated with complex mycobacterial diseases (Stucki and Gagneux, 2012). Various abnormalities like chromosomal insertions or deletions can be identified with more advanced microarray based comparative genomic hybridization (aCGH). CGH is a popular molecular cytogenetic technique for genome-wide screening of cells for chromosomal copy number variations. It uses two differentially labeled genomic DNAs: test and control sample which are simultaneously cohybridized to metaphase chromosomes. The differentially colored fluorescent signal intensity of the fluorophore labeled test DNA relative to control sample DNA is linearly plotted along the length of each chromosome to provide a cytogenetic representation of copy number variation between the two sources (Kallioniemi et al., 1992). However, CGH shows a very limited resolution of alterations of approximately 5–10 Mb (Kirchhoff et al., 1998; Lichter et al., 2000). To overcome this limitation, a more advanced high-resolution platform is known as array CGH (aCGH) has been developed. Instead of targeting metaphase chromosomes, it utilizes cloned DNA elements (known as probes) arrayed on a slide as the targets for analysis (Lucito et al., 2003). These probes are from different origins and vary in size like oligonucleotides (25–85 base pairs), bacterial artificial chromosomes (BACs; 80,000–200,000 base pairs). The probes used in aCGH are far smaller than the metaphase chromosomes which allows greater mapping resolution in aCGH than the traditional CGH. The mapping resolution depends upon both the probe size and genomic distance between DNA probes (Theisen, 2008).

The human genome project initiated in 1990 annotated the DNA sequence of the complete euchromatic human genome. Since then, the sequencing technologies [Sanger and next-generation sequencing (NGS)] have remained the hottest topic in the field of genomics research (Gasperskaja and Kućinskas, 2017). In the modern DNA sequencing era, with the ongoing technological advancement in the field of genomics, the sequencing technologies are revolutionizing the genome research especially with the high-throughput NGS (HT-NGS). It has a wide range of applications such as: chromatin immunoprecipitation (‘ChIP’) with DNA microarray (‘chip’) also known as ‘ChIP-on-chip’ and ChIp-sequencing (ChIP-seq) (Pareek et al., 2011).

Historically in 1975, the “first generation” DNA sequencing technique, known as ‘Sanger’s method’ or ‘dideoxy chain termination method,’ was developed based on specifically labeled chain terminating dideoxynucleotides (ddNTPs) incorporated by DNA polymerase during *in vitro* DNA synthesis. The fundamental principle behind this targeted sequencing technique is that the ddNTPs are different from dNTPs at 3′ carbon and fail to make phosphodiester bond with the next nucleotide which terminates the nucleotide chain elongation and hence replication halts. In this way, different bands of varying lengths are generated which are then separated on a polyacrylamide gel. After band separation, a laser reads the gel to detect the fluorescent intensity of each band in the form of colored peaks in a chromatogram. These colored peaks represent the nucleotide in that specific location in the DNA sequence (Russell, 2002).

Although Sanger method has proven useful in performing a thorough analysis of DNA, its use has been limited because of the high cost and size limitation. The Sanger method can only read short pieces of DNA (1000–1200 base pair) and the quality degrades after 700–900 base pairs. More recently, to overcome major stumbling blocks of first generation sequencing, new generations of sequencing techniques have been introduced which include NGS. NGS is capable of sequencing millions of DNA fragments through a massively parallel analysis with much reduced cost producing huge sequencing data. It has proven to be the new game changer for DNA sequencing. Although NGS exploits the principle similar to that of Sanger’s method of sequencing, which relies on the separation of labeled DNA elements by electrophoresis and identification of emitted signals, NGS uses array-based sequencing. It combines Sanger’s techniques (sequencing, separation and detection) for analysis of millions of samples in parallel at reduced cost with high throughput. It involves three steps: library preparation- small fragments of DNA created using random fragmentation (enzymatically or sonification) and ligated with custom linkers, amplification- done by PCR (emulsion PCR or bridge PCR), sequencing- DNA sequenced using “sequencing by synthesis” or “sequencing by ligation” (Zhang et al., 2011; Ari and Arikan, 2016). The ever growing field of sequencing has sparked an enormous range of applications of NGS technology in different research fields such as elucidation of the molecular basis of genetic diseases, infectious diseases and cancer (Del Vecchio et al., 2017).

ChIP assays are the most invaluable methods to identify the protein binding sites on DNA. ChIp-seq couples ChIP assays with NGS to investigate the genome-wide DNA binding sites for physical binding interactions of transcription factors. In ChIP-seq, formaldehyde fixation is used to irreversibly cross-link proteins to their bound DNA. The cross-linked chromatin is sheared with sonication or restriction enzymes to generate small fragments of DNA associated with a particular protein of interest followed by immunoprecipitation with desired antibody-bound magnetic beads. For NGS library preparation, the precipitated genomic DNA is used as input and is sequenced for DNA binding site analysis (Gasperskaja and Kućinskas, 2017). A more recent approach named ‘ChIP-on-chip’ combines ChIP with microarray analysis. In this method, the precipitated DNA fragments are hybridized to a microarray chip for analysis. It generates a global genome-wide chromatin maps depicting genome-wide binding sites of protein which may help to identify the functional elements in the complete genome. While this technique proved to be a revolutionary approach to study large genomic regions, it suffered from certain technical limitations such as high cost and requirement of a large amount of DNA thus extensive amplification leading to biasness and allelic variants hindered by cross-hybridization (Mikkelsen et al., 2007).

Hence, genomic analysis techniques provide an enormous amount of valuable information which may be translated in form of novel biomarkers to expedite antigen discovery. The genomic analysis usually begins with the identification and selection of potential coding regions. Along with this, attribution of functions to the selected novel proteins on the basis of sequence homology followed by a reverse genetic evaluation to characterize the complete repertoire of unannotated hypothetical proteins may be carried out (Geluk et al., 2014). Among the major mycobacterial infections, the complete genome sequence of *Mtb* H37Rv (Krogh et al., 2001) and CDC-1551 strains (Betts, 2002) and *M. bovis* AF2122/97 strain (Garnier et al., 2003) have revolutionized a big impact on the pace of anti-mycobacterial drug discovery. The genome sequence of *M. leprae* strain TN (Singh and Cole, 2011) has also been established. Using various *in silico* approaches, the whole set of protective antigens can easily be identified from the microbial pathogen’s genome without even cultivating it in the laboratory (Rappuoli, 2000). Hence, genome analysis can circumvent the laborious, costly and time-consuming conventional approaches and may pave the way to a better and faster discovery of antigenic targets against mycobacterial infections.

### Transcriptomics

The transcriptome reflects the set of all RNA molecules or transcripts in a cell or organism. Transcriptomics aim to study all species of transcripts including mRNAs, non-coding RNAs and small RNAs produced in a cell of an organism at a specific time (Wang et al., 2009; Kunnath-Velayudhan et al., 2017; Lowe et al., 2017). Transcriptomics analysis has played a central role in unraveling the gene expression during a particular physiological condition and deciphering the intricacies of regulations at the transcriptional level. Expression profiling of transcripts could be targeted to explore the specific genes which show expression or overexpression in host and pathogens simultaneously representing a complete atlas of hot-spots of host-pathogen interactions (Kaiser et al., 2004). Several technologies in the field of transcriptomics have emerged to derive and quantify the RNA content, including hybridization-based and sequence-based approaches. The dominant contemporary techniques like microarrays typically measure the transcripts by hybridization of fluorescently labeled cDNA against a custom-made array of complementary probes or high-density spotted oligonucleotide microarrays. The transcriptional profiling by hybridization-based approaches is labor saving with high throughput and reduced cost. However, these suffer from some limitations such as they can detect only known sequences, high background levels generally lead to cross-hybridization and interfere with detection. Although microarray technology continues to support transcriptomics research, the advent of sequence based approaches have dramatically expanded transcriptomics in the past few years (Wang et al., 2009).

In contrast to classical hybridization techniques, the high-tech sequencing based approaches directly determine the nucleic acid sequence of cDNA. In earlier times, Sanger’s method was used to sequence cDNA or EST libraries (Gerhard et al., 2004), but this method was expensive with relatively low throughput. To overcome this, high throughput tag-based transcriptome profiling methods were developed which included cap analysis gene expression (CAGE), serial analysis gene expression (SAGE), and massively parallel signature sequencing (MPSS). Since, these methods were based on conventional Sanger sequencing technique, these were expensive and failed to map some of the short tags to the reference genome. Additionally, they failed to analyze transcript isoforms which are generally indistinct from each other. These limitations reduced the potential use of conventional sequencing technology as transcriptome profiling method (Wang et al., 2009).

Recently, the newly developed high-throughput DNA sequencing techniques have enabled highly sensitive analysis for mapping, profiling and quantifying RNAs. This rapidly growing transcriptome profiling technique is known as RNA-Seq or whole transcriptome shotgun sequencing (WTSS). RNA-Seq utilizes an NGS platform and is replacing gene expression microarrays at a high rate. For this method, RNA (fractionated or total) is first converted to cDNA molecules with the help of reverse transcriptase followed by PCR amplification. Each molecule is then sequenced using NGS sequencing platform. Following sequencing, a genome-scale transcription map is generated when the output reads are aligned to reference transcripts or reference genome (Wang et al., 2009). RNA-Seq is an effective and excellent approach for transcriptome profiling of host and pathogen simultaneously. Moreover, this technique has also been successfully used to compare HCV- or HIV-infected T-cells to uninfected T-cells *in vitro.* It has revealed differentially expressed transcripts of the virus and the metabolic effects of viral infection on the target cells (Lefebvre et al., 2011).

Exploiting the above mentioned transcriptomic techniques, a number of studies have been reported describing the identification of various RNA molecules involved in different regulatory networks responsible for the virulence of pathogenic mycobacterial species. RNA-Seq and high-density tiling arrays have deciphered a large repertoire of previously unknown non-coding mycobacterial RNA including novel antisense transcripts, 5’ and 3’ untranslated regions and intergenic small RNAs (sRNAs) (Arnvig and Young, 2012; Michaux et al., 2014).

Non-coding RNA (ncRNA) molecules represent RNA transcripts that are generally not translated into a protein. Although, exceptionally, some ncRNA may contain an ORF and may translate into a polypeptide chain. There are different classes of ncRNA defined on the basis of cellular processes such as ncRNAs involved in mRNA translation (rRNAs and tRNAs), splicing (small nuclear RNAs -snRNAs), modification of rRNAs (small nucleolar RNAs-snoRNAs) and gene expression regulation (microRNAs-miRNAs, piwi-interacting RNAs-piRNAs, long non-coding RNAs-lncRNAs (Arnvig and Young, 2012; Qureshi and Mehler, 2012).

The sRNAs are generally the non-coding small transcripts in the range of 50–250 nucleotides in length. They are involved in gene silencing and post-transcriptional regulation and are generally encoded opposite the ORF (*cis*-encoded) or between ORF (*trans*-encoded) (Haning et al., 2014). The first mycobacterial stress regulatory sRNA was identified in 2009. The cDNA libraries of low molecular weight *Mtb* transcriptomes (exponential and stationary phase) were analyzed to identify 5 *trans*-encoded and 4 *cis*-encoded sRNAs in *Mtb* H37Rv (Arnvig and Young, 2009). Until now, a total of nearly 200 sRNAs have been identified in *Mtb* (Gerrick et al., 2018). The sRNAs discovered so far have gained significant attention, especially in pathogens as regulators of transcription factors, pathogenic genes, outer membrane adaptation to stress conditions like the variation in environmental pH, temperature and anaerobic stress (Haning et al., 2014; Michaux et al., 2014).

miRNAs are evolutionarily conserved small non-coding RNA molecules of 20–24 nucleotide length. These have been reported to play a regulatory role at the post-transcriptional level by binding to the 3’-UTR of their target mRNAs and inhibiting their translation. In pathogenic mycobacterial species, these miRNAs have been demonstrated to play an important role as immunomodulators by regulating the genes expressed by immune cells of the host and in-turn supporting its growth and survival inside the host. In recent studies, it has been shown that the innate immune response generated against TB is regulated by these miRNAs. Additionally, miRNAs differential expression during TB reflects disease progression and are capable of distinguishing active TB from latent TB (Palazzo and Lee, 2015; Ahluwalia et al., 2017; Sabir et al., 2018).

Hence, the uniquely expressed RNAs identified by high-throughput transcriptomic methods provide new insights into pathogenesis and could be targeted as potential biomarkers or as therapeutic agents against mycobacterial diseases.

### Proteomics

Proteome reflects the entire set of expressed proteins in a cell, tissue or organism at any given time (Theodorescu and Mischak, 2007). Proteomics covers a number of different aspects of protein function, including structural proteomics: large-scale analysis of protein structures, expression proteomics: large-scale analysis of protein expression and interaction proteomics: large-scale analysis of protein interactions. The main aim of proteomics is to study and characterize the information flowing within a cell or organism in the form of protein pathways and networks, (Petricoin et al., 2002) in order to understand the functional importance of proteins (Vlahou and Fountoulakis, 2005). Proteomics studies provide a deep understanding of the various virulent factors in different disease causing microorganisms and can aid the discovery of suitable markers as novel therapeutic agents (Fournier and Raoult, 2011).

Conventionally, different chromatographic methods have been used for purification and separation of proteins such as gel filtration/size exclusion chromatography (SEC), ion exchange chromatography (IEC) and affinity chromatography (Jungbauer and Hahn, 2009; Voedisch and Thie, 2010; Hage et al., 2012). To analyze selective proteins, techniques like western blotting and ELISA have been widely used. Sodium dodecyl sulfate-polyacrylamide gel electrophoresis (SDS-PAGE), two-dimensional gel electrophoresis (2-DE) and two-dimensional differential gel electrophoresis (2D-DIGE) techniques have also been used to separate complex protein samples (Marouga et al., 2005; Issaq and Veenstra, 2008). An emerging proteomics technique, named as protein microarrays or protein chips provides a versatile platform to analyze proteins on large scale. While mass spectrometry, another analytical technique, is used to analyze complex protein mixtures on the basis of the mass-to-charge ratio of charged particles with high sensitivity (Yates, 2011). Additionally, Edman degradation is used to sequence amino acids in a particular protein (Smith, 2001). To quantify global changes in protein numbers, a number of peptide quantitation techniques have been developed including, metabolic based labeling [stable isotope labeling with amino acids in cell culture (SILAC)] and isotope-coded affinity tag (ICAT) labeling, isobaric mass tagging [isobaric tag for relative and absolute quantitation (iTRAQ)], chemical and enzymatic derivatization [quantitation by isobaric terminal labeling (QIRT)] (Ong and Mann, 2006; Shiio and Aebersold, 2006; Wiese et al., 2007; Kroksveen et al., 2015) etc. The three-dimensional structures of proteins are obtained using two popular experimental high-throughput techniques: nuclear magnetic resonance (NMR) spectroscopy and X-ray crystallography (Smyth and Martin, 2000; Aslam et al., 2017).

With the advent of proteomics techniques, their applications have been wide-ranging and expanded in almost every discipline of biological sciences. *In silico* analysis of the available proteomic data has defined several new ‘omes’ having potential antigenic targets. These include the exportome (Van Ooij et al., 2008), surfome (Sargeant et al., 2006), and interactome (Sanchez et al., 1999). The surfome or surface proteome of several pathogens has been identified using proteolytic shaving (Rodríguez-Ortega et al., 2006) and biotinylation (Cullen et al., 2005). Currently available proteomic techniques exploiting peptide libraries and antibody microarrays have been used to analyze *Mtb* proteome to identify potential antigen candidates (Kunnath-Velayudhan and Porcelli, 2013). There was a report where workers have annotated most potential subunit vaccine candidates by comparing the mycobacterial proteomes of *Mtb* and *M. bovis* BCG. They observed that Rv3407, a DNA vaccine candidate could be used to improve the overall efficacy of the existing BCG vaccine (Mollenkopf et al., 2004). Others have also discovered novel antigenic markers from the identified secreted and transmembrane proteins employing proteomics approach- glutathione S-transferase (GST) fusion protein purification strategy (Zhou et al., 2015). Similarly, *Mtb* Rv0444c, Rv3692, and Rv2031c have been identified as possible candidate biomarkers from an analysis performed through MALDI-TOF-MS (Zhang et al., 2012). These may be targeted for the development of diagnostic assays against TB in the near future.

### Metabolomics

In the present “omics” era, metabolomics is rapidly emerging as a field of science to study the systematic identification, quantification and analysis of cellular metabolites within a given biological system (cell, tissue, organ, biological fluid or organism) at any given time. It is a collection of sophisticated analytical techniques to study the outcome of complex networks of biochemical reactions providing an understanding of the cellular physiology on a global biochemical scale (Mirsaeidi et al., 2015; Nandakumar et al., 2015).

Some of the modern analytical platforms used to study metabolite profiles include proton nuclear magnetic resonance (1H-NMR) spectroscopy, gas chromatography-mass spectrometry (GC-MS) and liquid chromatography-mass spectroscopy (LC-MS). These have been used to provide sensitive and reliable detection of metabolites to be exploited in diagnosis and prognosis of several infectious diseases (Weiner et al., 2012; Ghannoum et al., 2013; Mickiewicz et al., 2014). The metabolomics studies of mycobacterial pathogens are still in their nascent period of development. The recent studies about *Mtb* metabolome have provided unique insights into the biochemical composition, organization, activity and regulation of its physiological network (Nandakumar et al., 2015). The metabolites arising from a mycobacterial pathogen or its host have yielded important information describing undefined metabolism and pathogenic characteristics linked to the pathophysiology of mycobacterial infections (Miyamoto et al., 2016). du Preez and Loots have analyzed the sputum of 34 TB patients with 2D-gas chromatography time-of-flight mass spectrometry (GC-MS) (du Preez and Loots, 2013). They successfully identified 22 metabolites (14 *Mtb* metabolites and 8 host-related metabolites) as potential biomarkers against TB (du Preez and Loots, 2013). Similarly, in another study, using the same analytical tool, it was reported that 2-acetylamino-2-deoxy-b-D-glucopyranose, a-L-mannopyranose and D-galactose-6-deoxy could be targeted to differentiate TB infected patients from non-infected persons (Cha et al., 2009; O’Sullivan et al., 2012). In a different liquid chromatography-mass spectrometry (LC-MS) based metabolomics study, it was observed that rpoB mutations change the *Mtb* metabolic profile and it plays an important role in its metabolism. A total of 99 molecular features were found different in the *Mtb* rifampin-resistant strains (Bisson et al., 2012). In a different study, non-targeted ultrahigh-pressure liquid chromatography time-of-flight mass spectroscopy (UPLC-TOF-MS) was exploited to distinguish a cohort of patients infected with leprosy having bacterial index < 1 from those with a bacterial index > 4 (increased metabolites: polyunsaturated fatty acids, eicosapentaenoic acid and docosahexaenoic acid) (Al-Mubarak et al., 2011).

Compared to the other ‘omics’ technologies, metabolomics has fewer limitations and offers potential advantages in terms of specificity and sensitivity (van Ravenzwaay et al., 2007). As metabolomics captures the snapshot of the metabolic status of the genes providing useful insights about the biochemical networks under study, it allows more complete understanding of cell functions perhaps far more than genomics, transcriptomics or proteomics can (Lindon et al., 2003).

### Reverse Vaccinology (RV)

**FIGURE 2:**
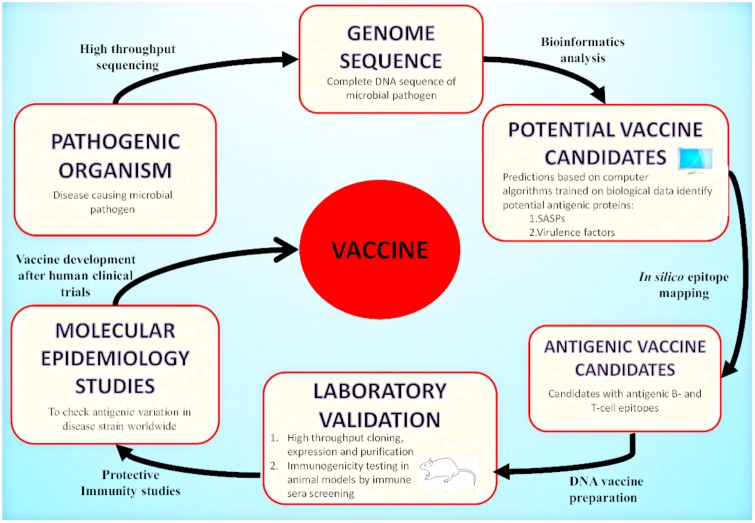
Reverse Vaccinology approach: A schematic representation of vaccine development by RV is illustrated in the presented flowchart. RV starts with the computational analysis of the complete genome sequence of the targeted pathogenic organism. Computational predictions are based on algorithms trained on biological data obtained from experimentally carried out studies. The potential vaccine candidates include surface associated and secretory proteins (SASPs) and virulence factors. These are further evaluated to identify protein candidates with antigenic epitopes for B-cells and T-cells. These proteins are then amplified by PCR and expressed in suitable vectors. The recombinant proteins produced are purified and used for immunogenicity testing in animal models (mice). Based on immune sera screening (FACs, Serum Bactericidal Activity), the recombinant proteins capable of inducing sera bactericidal antibodies are selected. The top candidates enter the pre-clinical stage of vaccine development. After the molecular epidemiological studies, the best candidates are used for clinical trials in adults, adolescents and infants and finally they enter the vaccine formulation process.

Today, with the advent of genomic technology, the genome-based antigen selection is possible and allows the discovery of antigen and vaccine design. One approach that mines pathogenic bacterial genomes for antigen discovery is known as “Reverse Vaccinology” (RV). RV has emerged as an effective strategy that uses bioinformatics techniques with the aim to identify highly protective and immunogenic peptides encoded by immunologically exposed pathogenicity factors by screening the entire genomes of microbial pathogens (Movahedi and Hampson, 2008; Seib et al., 2012; Donati and Rappuoli, 2013; Figure 2). RV based antigen discovery pipeline involves genome sequence analysis for the identification of antigenic proteins (surface exposed or secreted) expressed by the pathogen, their cloning and expression followed by synthetically producing each protein. The best selected candidates could be tested in the clinical trials for validating their immunogenicity after *in vitro* immunogenicity examination in cells and animal models. The identified antigens may be targeted for vaccine discovery. To date, RV has been targeted to devise universal and effective vaccines against bacterial pathogens for which the discovery of vaccines was previously impossible. Among these, *N. meningitidis* serogroup B (MenB) (Pizza et al., 2000), against which there was no effective vaccine, was the first pathogen targeted for the development RV based human vaccine (Delany et al., 2013; Rappuoli et al., 2016).

With the help of RV, whole-genome studies are now being more focused on the development of target specific epitope-based vaccines. An epitope or an antigenic determinant is the specific part of antigen interacting with the immune system (T-cell, B-cell and antibodies). The antigenic epitopes elicit an immune response by interacting with the CD8+ T immune cells and CD4+ T immune cells and may be used in ‘reverse’ to target novel antigens (Sette and Rappuoli, 2010). The immune cells- B and T lymphocyte play a major role in antigen recognition and elicitation of the immune system. The B lymphocytes are the plasma cells that produce antibodies when a foreign antigen triggers immune system and function as humoral immunity component of the adaptive immune system. The epitopes of the antigens are identified by the paratopes of antibody. The T lymphocytes play a central role in cell-mediated immunity. Hence, the prediction of the immunodominant T and B cell epitopes plays an important role in the determination of the peptide-based candidate vaccines (Kanampalliwar et al., 2013).

Based on RV, a number of web-based programs have also been developed to assist the scientific community in identifying potential vaccine candidates against mycobacterial infections. These include MycobacRv, Violin, VaxiJen, and MtbVeb, etc. MycobacRv is an RV based database of potential mycobacterial adhesins vaccine candidates from 23 strains and other species of mycobacteria. It houses detailed epitope information from the predicted adhesins and surface-localized/extracellular proteins which may further facilitate the development of epitope-based mycobacterial vaccines (Chaudhuri et al., 2014). Vaccine Investigation and Online Information Network (VIOLIN) is another web-based database that integrates vaccine literature mining, vaccine data curation and storage. It also provides an analytical platform for potential vaccine target prediction against various infectious agents (He et al., 2014). Likewise, VaxiJen is another useful resource available online for the prediction of protective antigens and subunit vaccines. The predictions are alignment independent and solely based on the physicochemical properties of the target proteins (Doytchinova and Flower, 2007). MtbVeb is a comprehensive database for designing novel vaccines against 59 existing and emerging *Mtb* strains employing antigen, strain and epitope based approaches (Dhanda et al., 2016). A growing number of studies reporting antigen identification published in the literature have provided valuable insights into RV based vaccine research. Some of them have been discussed in the coming sections of this review paper.

### Challenges Faced by Contemporary ‘Omics’ Approaches During Antigen Discovery

The available high-throughput ‘omics’ approaches have made it possible to identify potentially important biomarkers in various microbial pathogens in a much smaller time than the conventional approaches. The wide availability of data generated by these ‘omics’ technologies offer ample opportunities to unravel the disease mechanisms but also present the scientific community with significant challenges to extract the knowledge from such huge data and its application for the welfare of the society.

In genomics, the pathogenic microorganisms with larger genomes, that fails to be cultured *in vitro* or if there are no animal models available, may not be suitable for antigen discovery utilizing RV because of the huge number of possible targeted proteins with unknown function (Schussek et al., 2014). In the case of transcriptomics, the information generated from deep sequencing studies need *in vivo* validation and also require validation for multiple isolates of the microbial pathogen (Schussek et al., 2014).

Similarly, although proteomics offers advantages in antigen discovery, it still suffers from certain limitations. While performing proteomics analysis, the organism is allowed to grow in highly favorable conditions (*in vitro)* and is generally isolated at a specific phase of the cell cycle which certainly does not depict the *in vivo* environment of that organism (Singh et al., 2015). Furthermore, the proteomics studies may not be suitable enough to identify protein complexes which are resistant to proteases as reported earlier for pili associated proteins, which have been demonstrated as potential vaccine candidates for *Staphylococcus aureus* and *Streptococcus pneumonia* (Schussek et al., 2014). Moreover, the proteomics approach gives a limited level of understanding of the protein level events of microorganisms since the mRNA transcription of a gene necessarily does not give an estimation of its translated protein level. The reason could be: the transcribed mRNA might degrade quickly or it might get translated into protein ineffectively or alternative splicing might result in the generation of multiple proteins. Another reason could be the post-translational modifications of proteins which might result in an inactive protein (Kornblihtt et al., 2013). Another major limitation of the proteomics approach is many proteins are involved in complex formation to become completely functional (Srinivas et al., 2002). Additionally, the secondary and tertiary structures of proteins are often difficult to maintain during their analysis. These generally get denatured by the action of enzymes, heat or by external stress. The proteins of low abundance are often found difficult to detect as these cannot be amplified like DNA. Like in plasma, cytokines are present in very low quantity (1–5 pg/mL) and proteomics tools can analyze proteins mostly located at the higher end of the concentration spectrum. Hence, to study these low abundant proteins, the high abundant proteins are removed from plasma. However, this removal is often accompanied by the loss of several potentially important biomarkers resulting from co-removal of antigenically important proteins bound to the high-abundance proteins (Granger et al., 2005; Cho, 2007). For these reasons explained above, very often, the proteomics experiments performed in one laboratory are poorly reproducible in other laboratories.

Nevertheless, the metabolomics key features for several diseases (Monteiro et al., 2013; Aretz and Meierhofer, 2016) have been reported, the potential bottlenecks still exist at various levels of quality biomarker identification. It is hampered by the huge and dynamic variation in the metabolic levels between people, tissues and various time points. The other bio-molecular states like the genome, transcriptome and proteome are comparatively much more stable than the vastly fluctuating metabolites (Aretz and Meierhofer, 2016).

Hence, to fulfill the huge demand for novel robust biomarkers to curb the mycobacterial infections, different ‘omics’ platforms must together be integrated to reveal, assess and track down the novel molecular patterns reflecting the disease-perturbed networks.

## Application of Proteome-Scale *In Silico* Strategies for Discovering Potential Antigens

**FIGURE 3:**
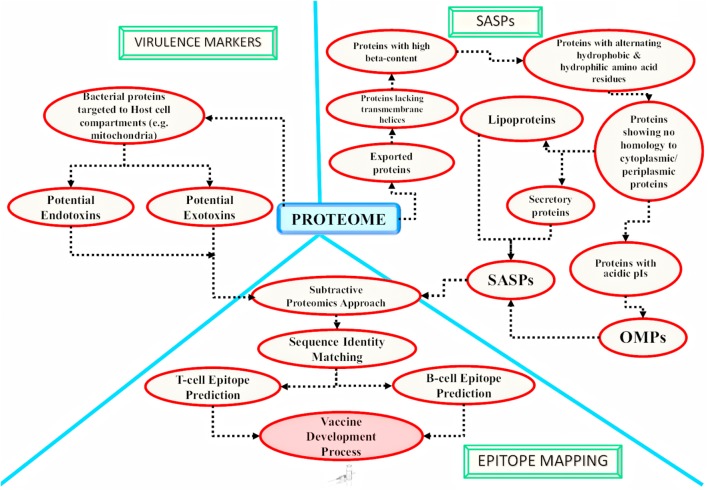
Application of *in silico* approaches for mycobacterial antigen discovery: a schematic overview of the methodologies currently followed using *in silico* approaches for mycobacterial antigen discovery is shown here. These antigens may be targeted for developing medical interventions against infectious agents. The bacterial factors targeting the host cell compartments are considered as established virulence factors and are reported to be involved in host cell ‘hijacking’ [as reported for mitochondria targeted *M. avium* subsp. *paratuberculosis* (MAP) proteins by Rana et al. (2015b)]. There are computational algorithms available which may further identify potential endotoxins and exotoxins from the potential host targeted proteins. The obtained host targeting proteins can further be subjected to epitope mapping analysis. On the other hand, the complete proteome of the pathogen can also be targeted for the identification of potential surface associated and secretory proteins (SASPs), which include lipoproteins, secretory proteins and Outer Membrane Proteins (OMPs). Epitope mapping may be carried out for the identified SASPs (Rana et al., 2015c). The screened epitopes might be utilized for developing next generation vaccines [e.g., chimeric multi-subunit artificial model vaccine as reported by Rana et al. (2016) and novel serodiagnostic markers]. Similar *in silico* studies may be targeted to identify novel potential antigens against other infectious agents also.

A number of computational programs exploiting bioinformatics algorithms have been made available for the genome/proteome sequence retrieval, sub-cellular localization of proteins on the basis of the presence of special protein signature sequences (e.g., secretory signal peptide, transmembrane helices, lipoprotein signal peptide, etc.), structural prediction, epitope mapping, virulence prediction and potential vaccine development. Some of the commonly used programs and databases have been summarized in Table 3. By utilizing such tools, numerous *in silico* studies have reported results deciphering the surface associated and secretory proteins (SASPs) such as OMPs, lipoproteins and secretory proteins. These are the most exposed proteins and may serve as virulence factors for the pathogens (Rana et al., 2014, 2015a,b; Rana and Akhter, 2016). These reports also demonstrate epitope mapping to target the most suitable potential antigens for vaccine development (Figure 3; Fournier and Raoult, 2011; Rana et al., 2014, 2015a,b, 2016; Rana and Akhter, 2016). In the next subsections, we have summarized the utility of the proteome-scale *in silico* screening strategies based on computational programs (Table 3), to identify the virulence determinants and antigenic targets in microbial pathogens.

**Table 3:** Commonly used software programs and databases for *in silico* approaches in antigen discovery.

Program/Database	Description	Features	Limitations
**(A) Genome proteome retrieval**			
NCBI (Agarwala et al., 2018)	(1) Retrieval of genome and proteome data	(1) Automated system for storing and retrieval of biomedical and genomic information in form of databases and software.	Redundancy in genomic information.
	(2) Available at: https://www.ncbi.nlm.nih.gov	(2) Data stored is open access and powerful data analysis and retrieval tools are available.	
		(3) Quick links to several other tools are available on the web portal.	
		(4) It produces information in accessible formats.	
Integrated Microbial Genomes and Microbiomes (IMG) (Markowitz et al., 2012)	(1) Comparative analysis of publicly available genomes	(1) Employs NCBI’s References Sequence database as its main source of genomic data and ‘primary’ annotations consisting of predicted genes and protein products.	Coherence of functional annotations.
	(2) Available at: https://img.jgi.doe.gov	(2) For every gene, a list of ortholog, paralog, and homolog based on sequence similarities is provided.	
		(3) The protein coding genes are computed using NCBI BLASTp and RNA genes using BLASTn.	
		(4) It identifies gene fusions and conserved gene cassettes in the form of putative operons to be used in data integration pipeline.	
		(5) Genomes compared at two levels- gene content and functional capabilities.	
		(6) IMG follows rigorous tool maintenance and content update procedures.	
Biomartr (Drost and Paszkowski, 2017)	(1) Genomic data retrieval	(1) Handles multiple genomes simultaneously.	Requires programming expertise.
	(2) Available at: https://github.com/HajkD/biomartr	(2) Assigns Gene Ontology information and sequence homology relationships among different microbial organisms.	
		(3) Genomic data retrieval and functional annotation is fully annotated and easy to use.	
		(4) It offers a high degree of clarity, transparency and reproducibility of analyses.	
Gene Locator and Interpolated Markov Modeler (GLIMMER) (Delcher, 1999; Kelley et al., 2012)	(1) Open reading frames prediction	(1) Identifies the coding regions by utilizing interpolated Markov models (IMMs).	Ineffective on metagenomic sequences, computationally expensive.
	(2) Available at: http://www.cs.jhu.edu/$\sim$genomics/Glimmer/	(2) Distinguishes coding from non-coding DNA.	
		(3) Identifies long-ORFS and trains all the six IMMs of both coding and non-coding DNA.	
Artemis (Carver et al., 2012)	(1) Genome browser and functional annotation	(1) Memory-based approach for visualizing and analyzing large datasets.	Provides a limited set of analyses.
	(2) Available at: https://www.sanger.ac.uk/science/tools/artemis	(2) Supports various file formats including BAM, VCF, BCF and FASTA.	
		(3) Displays multiple different read alignment views of the same dataset at once which can be compared across different genomes simultaneously.	
		(4) It can visualize and analyze data from different sequencing technologies.	
**(B) Protein localization**			
TargetP1.1 (Emanuelsson et al., 2000)	(1) Mitochondrial-targeting proteins	(1) Localizes different proteins on basis of N-terminal presequences: mitochondrial targeting peptide (mTP), chloroplast transit peptide (cTP) and secretory pathway signal peptide (SP).	Only Limited number of protein sequences (2000) can be submitted at a time, poor discrimination between mTPs and cTPs.
	(2) Available at: http://www.cbs.dtu.dk/services/TargetP/	(2) Neural network-based tool.	
		(3) User can choose cutoffs for predictions and hence provides more specific output.	
TATFIND (Rose et al., 2002; Wagley et al., 2014)	(1) Predicts the presence of prokaryotic Twin-Arginine Translocation (Tat) signal peptides	(1) Differentiates the structurally similar signal sequences of sec and tat substrate types.	Proteins lacking Tat signal sequence that can be transported by the Tat system in a ‘hitchhiker’ fashion cannot be predicted.
	(2) Available at: http://signalfind.org/tatfind.html	(2) Prediction is based on assigned putative Tat substrates signal sequences with high accuracy.	
SignalP (Nielsen et al., 1997)	(1) Signal peptide prediction	(1) Predicts cleavage sites based on a combination of several artificial neural networks.	Low precision while discriminating cleaved signal peptides and uncleaved N-terminal signal-anchor sequences.
	(2) Available at: http://www.cbs.dtu.dk/services/SignalP/	(2) It shows high performance and can easily be applied to genome-wide data sets.	
TMHMM (Krogh et al., 2001)	(1) Transmembrane domains prediction	(1) Based on Hidden Markov Model approach.	Accuracy drops when signal peptides are present.
	(2) Available at: http://www.cbs.dtu.dk/services/TMHMM/	(2) Predicts transmembrane helices in proteins.	
		(3) Specialized modeling of membrane proteins.	
		(4) Offers both web-based version and a standalone version.	
		(5) Correctly predicts 97–98% of transmembrane helices and discriminates between soluble & membrane proteins with high specificity and sensitivity.	
LipoP (Sutcliffe and Harrington, 2004)	(1) Lipoprotein signal peptide prediction	(1) Discriminates lipoprotein signal peptides from other signal peptides and n-terminal membrane helices (in Gram-negative bacteria).	Limited number of protein sequences (5000 – 500,000) can only be submitted per submission, small protein sequences less than 70 and large protein sequences more than 5,000 amino acids cannot be submitted.
	(2) Available at: http://www.cbs.dtu.dk/services/LipoP/	(2) Identifies signal peptide I, signal peptide II and n- terminal transmembrane helix with high accuracy.	
		(3) Web version and Linux standalone is available.	
MitoProt (Claros and Vincens, 1996)	(1) Mitochondria signal peptide prediction	(1) Identifies the N-terminal Mitochondrial Targeting Sequence and cleavage site.	Fails to recognize proteins lacking targeting peptide sequences (mitochondrial outer and inner membrane proteins and transmembrane proteins).
	(2) Available at: https://ihg.gsf.de/ihg/mitoprot.html	(2) A discriminant function defined on the basis of physicochemical properties provides higher success rate.	
		(3) Maximal hydrophobicity of each hydrophobic face is calculated by averaging the total hydrophobicity weight of neighboring residues of a helical structure.	
SecretomeP (Bendtsen et al., 2004)	(1) Non-classical secretory proteins prediction	(1) Neural network based method.	Time consuming when handling large amount of data.
	(2) Available at: http://www.cbs.dtu.dk/services/SecretomeP/	(2) Identification on basis of specific chemical and biological properties.	
		(3) Uses TMHMM and PSIPRED to identify secreted proteins.	
		(4) Discriminates cytoplasmic proteins from classical secretory proteins.	
		(5) Identifies both gram positive and gram negative secreted proteins.	
**(C) Structure prediction**			
RaptorX (Källberg et al., 2012, 2014)	(1) Remote homology detection, protein 3D modeling, binding site prediction	(1) Exploits a non-linear context-specific alignment potential and probabilistic consistency algorithm.	Insufficiently cover several structures and sequence databases, poor secondary structure prediction accuracy if the input sequence fail to have a sufficient number of sequence homologs in the non-redundant database, limited domain prediction.
	(2) Available at: http://raptorx.uchicago.edu	(2) Assigns various scores to indicate the quality generated 3D protein model: GDT (global distance test) and uGDT (un-normalized GDT) for the absolute global quality, *P*-value for global quality and modeling error for each residue.	
		(3) It is very fast and detects even remotely related template sequences.	
MODELLER (Wallner and Elofsson, 2005; Webb and Sali, 2016)	(1) Comparative protein structure modeling	(1) Implements comparative protein structure modeling by satisfaction of spatial restraints.	Fails to model long insertions during loop modeling, at low (<50%) sequence identities performance drops.
	(2) Available at: https://salilab.org/modeller/	(2) Depends upon the alignment of the query sequence with the template protein (solved protein structure in PDB).	
		(3) *Ab initio* structure prediction of loop regions of proteins based on optimization based approach.	
		(4) It offers minimal violation of the spatial restraints while model building.	
Phyre (Kelley et al., 2015)	(1) Protein structure prediction	(1) Remote template detection, alignment, 3D modeling, multi-templates, ab initio.	Unable to accurately determine beyond the estimated position of a side chain the wider structural impact of a point mutation, the relative orientation of domains are predicted with low accuracy in *ab initio* modeled structures.
	(2) Available at: http://www.sbg.bio.ic.ac.uk/phyre2/html/page.cgi?id=index	(2) Uses hidden Markov models via HH search which improves the accuracy of alignment and detection rate.	
		(3) Incorporates Poing- a new *ab initio* folding simulation to model proteins regions lacking detectable homology.	
		(4) It is easy to use and can predict the phenotypic effect of a point mutation.	
		(5) It has ‘intensive’ mode for proteins who lack similar templates.	
**(D) Epitope prediction**			
Immune Epitope Database and Analysis Resource (IEDB) (Fleri et al., 2017)	(1) Database of experimentally characterized T- and B-cell epitopes	(1) Data repository offers experimental data characterizing T-cell epitopes and antibodies in humans, non-human primates and other animal species.	Very few animal species are available for analysis at IEDB.
	(2) Available at: https://www.iedb.org	(2) Epitopes involved in infectious disease, autoimmunity, transplant and allergy are also included.	
		(3) Provides different tools to analyze immune epitopes.	
		(4) It is easy to use and regularly updated.	
NetMHCIIpan (Karosiene et al., 2013; Nielsen and Andreatta, 2016)	(1) Prediction of peptide-MHC class I binding	(1) Based on artificial neural networks.	Low predictive performance.
	(2) Available at: http://www.cbs.dtu.dk/services/NetMHCIIpan/	(2) Uses MHC binding pocket pseudo sequence together with the peptide sequence as an input.	
		(3) Trained on 50,000 quantitative peptide-binding measurements including HLA-DR, HLA-DP, HLA-DQ and two mouse molecules.	
		(4) It shows high performance in comparison to other available methods and is capable of giving predictions to molecules not yet characterized experimentally.	
NetMHCpan (Hoof et al., 2009)	(1) Prediction of peptide-MHC class I binding	(1) Based on artificial neural networks.	Achieves low predictive performance for alleles like HLA-B molecules.
	(2) Available at: http://www.cbs.dtu.dk/services/NetMHCpan/	(2) Trained on the hitherto large set of MHC binding data, including HLA-A, HLA-B, MHC class I molecules of chimpanzee, gorilla, rhesus macaque and mouse.	
		(3) It shows accurate binding predictions to uncharacterized HLA molecules (HLA-C, HLA-G, chimpanzee and macaque MHC class I molecules) and high performance for non-human primates.	
ElliPro suite (Ponomarenko et al., 2008)	(1) B-cell epitope prediction	(1) Implements a modified version of Thornton’s method together with MODELLER program and Jmol viewer and residue clustering algorithm.	Fails to discriminate epitopes from non-epitopes efficiently.
	(2) Available at: http://tools.iedb.org/ellipro/	(2) Predicts and visualizes antibody epitopes in protein sequences and structures with high specificity.	
		(3) Implements three algorithms: (i) approximate of protein shape as an ellipsoid; (ii) calculate the residue protrusion index (PI); and (iii) cluster the neighboring residues based on their PI values.	
		(4) It is more advanced than Thornton’s method and considers each residue’s center of mass rather than its Cα atom.	
**(E) Endotoxin/Exotoxin prediction**			
Database for Bacterial ExoToxins (DBETH) (Chakraborty et al., 2011)	(1) Database of bacterial toxins	(1) Data repository of structure, sequence, interaction network and analytical results of 229 toxins from 26 bacterial genuses.	A number of other important bacterial toxins are not available for analysis at DBETH.
	(2) Available at: http://www.hpppi.iicb.res.in/btox/	(2) Prediction based on- homology with known toxin sequences/domains or specific bacterial toxin features classified using a support vector based machine learning techniques.	
		(3) Developed on CGI-PERL web based architecture.	
BTXpred (Saha and Raghava, 2007)	(1) Endotoxin or Exotoxin prediction	(1) Trained on a non-redundant dataset of 150 bacterial toxins (73 endotoxins and 77 exotoxins).	Number of other important bacterial toxins are not available for analysis at BTXpred.
	(2) Available at: http://crdd.osdd.net/raghava/btxpred/index.html	(2) Based on support vector machines modules for predicting bacterial toxins and discriminating exotoxins and endotoxins.	
		(3) Exotoxins sub-classified utilizing hidden Markov models, PSI-BLAST and a combination of the two.	
		(4) It provides fully automated annotation of genomic data.	
		(5) It has an option of predicting toxins either on the basis of an amino acid or dipeptide composition or PSI-BLAST.	
		(6) It allows users to predict functions of exotoxins using PSI-BLAST and HMM methods.	


### *In silico* Analysis for the Detection of Virulence Markers

Virulent factors represent the molecules essential for the growth of microbial pathogens which allow them to succeed and establish disease inside the host (Rana et al., 2015b). Earlier, the pathogenicity of bacteria was reported to be linked to toxins (Peterson, 1996) but later, it was considered to originate from the presence of various virulence determinants (Smith, 2003). Thus, it was concluded that targeting these potentially virulent factors would stop the disease establishment and would enable a rapid development of novel vaccines, antibiotics and new screening tests. The three main approaches that have been used for the identification of virulence genes from the complete genome involves: homology search with the experimentally characterized virulent factors (Rana et al., 2015c), identifying genes located in different pathogenic genomic islands (Akhter et al., 2007, 2008, 2012; Che et al., 2014) and the third approach involves identification of the virulence genes by genome comparison of strains having different pathogenicity profiles (virulent *versus* avirulent strains). Using an *in silico* approach, a set of 189 putative vaccine candidates have been identified from the complete *Mtb* genome (3989 gene products) (Zvi et al., 2008). A total of 40 promising therapeutic targets were identified in *M. abscessus* using novel hierarchical *in silico* approach and these may be exploited for novel drug discovery (Shanmugham and Pan, 2013). In an another *in silico* study performed on *Mtb*, 99 putative lipoproteins, playing important role in virulence, were identified using various bioinformatics utilities like TrEMBL database (Boeckmann et al., 2003), ScanProsite tool (Gattiker et al., 2002), SignalP (Nielsen et al., 1997), and TMHMM program (Krogh et al., 2001; Sutcliffe and Harrington, 2004). Similarly, in 2 different studies performed *in silico*, a total of 48 lipoproteins in *Mtb*, 25 lipoproteins in *M. leprae*, 75 lipoproteins in *M. avium*, 97 lipoproteins in *M. marinum* and 61 lipoproteins in *M. smegmatis* were computationally identified utilizing LipoP (Juncker et al., 2003; Rezwan et al., 2007). The pathogenic proteins targeting the host cell compartments like host mitochondria during infection are also the most commonly targeted virulent factors. A number of *in silico* proteome-wide studies have reported the potential mitochondria targeting proteins of the microbial pathogens (Moreno-Altamirano et al., 2012; Forrellad et al., 2013; Rana et al., 2015b). Forrellad et al., computationally identified 19 mitochondria targeting proteins from *Mtb* H37Rv virulent strain by utilizing the MitoProt program (mitochondria targeting proteins prediction) (Claros and Vincens, 1996), PSORT II prediction algorithm (sub-cellular localization) (Horton and Nakai, 1997) and SignalP (signal peptide sequence prediction) (Nielsen et al., 1997; Moreno-Altamirano et al., 2012). In a similar *in silico* approach, we have reported 46 MAP proteins as potential host mitochondria targeting proteins by employing different bioinformatics algorithms in tandem (Rana et al., 2015b). Firstly, complete MAP proteome was screened to detect the signal peptide sequence utilizing program SignalP and the identified exportome was analyzed for mitochondrial import signal screened through MitoProt II, TargetP and TPpred program (Savojardo et al., 2014). 46 MAP mitochondria targeting proteins were successfully identified. Out of these, 20 MAP proteins were defined as putative endotoxins from DBETH database (Chakraborty et al., 2011) and 14 MAP proteins as exotoxins by BTXpred tool (Saha and Raghava, 2007) which may be acting as potential virulent factors involved in MAP pathogenicity (Rana et al., 2015b).

### *In silico* Analysis for the Detection of Secretory and Surface-Associated Proteins (SASPs)

A ‘secretome’ of an organism represents the total secretory proteins that are being released into the external milieu. This group of proteins is commonly known as excretory/secretory (ES) proteins and is important for the establishment of pathogenic infection within the host (Gomez et al., 2015; Rana et al., 2016). The SASPs include secretory proteins and surface-associated proteins like lipoproteins and OMPs. These SASPs are nowadays considered as promising targets for antigen discovery. These offer ample opportunities for the development of new therapeutic solutions against different clinical infections as the SASPs including ES proteins that are present at the interface of host-pathogen interaction and may also function as immune modulators of the host cells (Zagursky and Russell, 2001). They also help in the pathogen survival inside the host organism and act as virulence factors.

We have earlier reported novel and much advanced *in silico* approaches (Rana et al., 2014) for the proteome-wide identification of SASPs of MAP, *M. leprae* and *Mtb* (Rana et al., 2016). The approach exploits the cardinal sequence and structural features of SASPs from mycobacteria. The exportome of the MAP, *M. leprae* and *Mtb* was first identified employing Target P1.1 program followed by transmembrane helix prediction by TMHMM and HMMTOP program. The selected proteins were further analyzed for the presence of α helix and β sheet by utilizing the JPRED3 (Cole et al., 2008) program and amphiphilicity computation using Vogel and Jahnig algorithm (Vogel and Jähnig, 1986). Further, lipoproteins were predicted by PRED-LIPO (Juncker et al., 2003) program and sub-cellular localization of proteins was done using PSORTb followed by identification of non-classical secretory proteins employing SecretomeP program. The performed proteome-wide analysis identified 57 OMPs, 38 lipoproteins, 63 secretory proteins in the MAP; 19 OMPs, 17 lipoproteins, 11 secretory proteins in *M. leprae*; 36 OMPs, 47 lipoproteins and 49 secretory proteins in *Mtb*. Similar *in silico* studies have been conducted on various pathogenic genomes and proteomes to identify the repertoire of SASPs which represented novel candidates as virulence factors. These include: *Taenia solium* (Gomez et al., 2015), *Phytophthora infestans* (Raffaele et al., 2010), *Yersinia pestis* (Yen et al., 2007), *Xanthomonas citri* (Ferreira et al., 2016), *Coxiella burnetii* (Ferreira et al., 2016), and enteric pathogens including *Shigella* spp, *E. coli*, *Vibrio cholerae*, *Yersinia enterocolitica* (Hashmi et al., 2010), *Salmonella spp.*, and *Anaplasma marginale* (Palmer et al., 2012).

### *In silico* Analysis for Epitope Mapping

Epitope mapping is one of the keystone steps to be considered while designing an effective potent vaccine (Palmer et al., 2012). It has remarkable advantages over the long established conventional methods since it is the most cost effective, highly specific and competent strategy to generate a specific desired long lasting immunity in the host. It also helps to avoid unwanted autoimmune responses. With the advent of diverse bioinformatics tools, epitopes are nowadays can easily be mapped from the whole genomes of microbial pathogens by performing *in silico* analysis, without immediate reference to the peptide fragments origin. Several immunoinformatics methods have been employed for designing a highly efficient vaccine that must be capable of generating a protective B and T-cell immune response (Davies and Flower, 2007; Rana et al., 2016). Numerous vaccine related studies integrated *in silico* RV approach to discover putative vaccine candidates against diverse pathogens.

In case of mycobacterial infections, RV studies reported that sxL, PE26, PPE65, PE_PGRS49, PBP1 and Erp were the six proteins identified with antigenic epitopes from *Mtb*, that could be targeted to design novel and more efficient vaccines against TB (Monterrubio-López, 2015). Eight proteins (MAP2698c, MAP2312c, MAP3651c, MAP2872c, MAP3523c, MAP0187c and the hypothetical proteins MAP3567 and MAP1168c) were also identified with highly immunogenic epitopes in the MAP as potential vaccine candidates for studying antibody and cell-mediated immune responses within infected hosts (Gurung et al., 2012). In our previous work, we have integrated biological knowledge together with bioinformatics tools to design a much more advanced methodology pipeline for epitope mapping of the MAP (Rana et al., 2015b) and *M. leprae* OMPs (Rana et al., 2016). Moreover, our earlier studies reported 83 potential OMPs from a total of 4356 MAP proteins, out of which 57 MAP proteins were identified as a core set of putative OMPs (Rana et al., 2014). The identified OMPs were first analyzed to identify the host homologous proteins and proteins with significant similarity to closely related *Mycobacterium* taxa for excluding them to prevent any potential cross-reactivity using BLAST analysis. Further, the non-homologous proteins were subjected to immunoinformatic analyses for the prediction of T-cell (MHC I: artificial neural network approach) (Nielsen et al., 2003; Tenzer et al., 2005)**;** MHC II: consensus approach (Wang et al., 2008) and B-cell epitopes ElliPro suite (Ponomarenko et al., 2008). Similarly, RV has been successfully applied against various other pathogens for identification of suitable antigens for vaccine development such as *Dichelobacter nodosus* (Myers et al., 2007), *Pasteurella multocida* (Al-Hasani et al., 2007) and *Mtb* (Kundu et al., 2016).

## Conclusion

In the present post-genomic era, the discovery of novel antigens for vaccines and diagnostics has expedited with the easy accessibility of information about the complete set of different mycobacterial genes and proteins. This offers an enormous amount of knowledge for the development of immunotherapeutics. In particular, the available mycobacterial genomes complemented by state-of-the-art ‘omics’ approaches together with the *in silico* screening strategies symbolize promising tools to discover potential vaccine candidates and therapeutic targets in diverse pathogenic mycobacterial species. In the modern era, proteomics based approaches are becoming faster and affordable and have shown a significant potential to identify the highly antigenic bacterial SASPs. With the advancement of next-generation sequencing techniques, it is strongly believed that these techniques may shortly be used as standard approaches for the development of medical interventions against mycobacterial pathogens. This will enable the identification of constant and variable genomic regions from thousands of variants, serotypes and isolates recovered from *Mycobacterium* infected patients. Hence, integrating diverse approaches starting with the various computational studies including comparative genomics within the taxonomic class of the *Mycobacterium* based on the sequencing data, their epidemiological coverage, functional genomics data and immunoprotective capacities must be utilized to discover excellent mycobacterial antigenic targets. Therefore, presently it is highly important to bridge ‘omics’ fields that are involved in antigen discovery together with system scale *in silico* methods as a pre-screen and standardization of methods for the flow of information to the *in vitro*, *in vivo* and animal model immunoprotection studies of individually selected candidates after utilizing these high-throughput screening methods.

## Author Contributions

All authors listed have made a substantial, direct and intellectual contribution to the work, and approved it for publication.

## Conflict of Interest Statement

The authors declare that the research was conducted in the absence of any commercial or financial relationships that could be construed as a potential conflict of interest.
